# Hispidulin Ameliorates Endotoxin-Induced Acute Kidney Injury in Mice

**DOI:** 10.3390/molecules27062019

**Published:** 2022-03-21

**Authors:** Kiryeong Kim, Jaechan Leem

**Affiliations:** Department of Immunology, School of Medicine, Daegu Catholic University, Daegu 42472, Korea; immunedcu@naver.com

**Keywords:** endotoxin, sepsis, acute kidney injury, natural flavonoid

## Abstract

Lipopolysaccharide (LPS) is an endotoxin that plays a crucial role in septic acute kidney injury (AKI). Hispidulin is a natural flavonoid that possesses various biological activities. Recent studies have shown that hispidulin administration alleviates various inflammatory diseases in animal models. This study aimed to investigate the renoprotective effect of hispidulin on LPS-induced AKI. Male C57BL/6 mice were administered LPS (10 mg/kg) with or without hispidulin (50 mg/kg). Hispidulin administration attenuated renal dysfunction, histological alterations, and the upregulation of neutrophil gelatinase-associated lipocalin. This flavonoid also reduced cytokine production and Toll-like receptor 4 expression, inhibited nuclear factor-κB and mitogen-activated protein kinase cascades, and alleviated immune cell infiltration. The oxidation of lipids and DNA was also inhibited by hispidulin administration. This antioxidant effect of hispidulin was associated with the downregulation of NADPH oxidase 4, the activation of catalase and superoxide dismutase activities, and the restoration of glutathione levels. Moreover, hispidulin administration attenuated tubular cell apoptosis by inhibiting caspase-3 pathway. These data suggest that hispidulin ameliorates endotoxin-induced kidney injury by suppressing inflammation, oxidative stress, and tubular cell death.

## 1. Introduction

Sepsis is a life-threatening response to infection and is a major cause of acute kidney injury (AKI) in critically ill patients [1]. Septic AKI is closely associated with poor clinical outcomes, including death and a longer duration of hospitalization [1,2]. Currently, the management of septic AKI consists of the rapid administration of antibiotics, adequate fluid resuscitation, and vasopressor therapy [2]. However, the current therapy is nonspecific and reactive; thereby, the development of novel therapeutic agents for patients suffering from septic AKI is warranted.

Accumulating evidence suggests that damage-associated molecular patterns (DAMPs) and pathogen-associated molecular patterns (PAMPs) play important roles in the pathogenesis of sepsis [2]. DAMPs are endogenous danger molecules that are derived from dead or dying host cells. These molecules can induce inflammatory responses by activating the innate immune system [2]. On the other hand, PAMPs are derived from microorganisms and drive inflammation in response to infections [3]. Among PAMPs, lipopolysaccharide (LPS) is an endotoxin found on the surface of Gram-negative bacteria [4]. The rodent model of endotoxin injection has been used extensively to study the pathogenesis of organ failure and to discover novel therapeutics [5]. This model mimics many clinical features of sepsis, but with no bacteremia. During sepsis, endotoxins are released into the bloodstream and transported to various organs [2]. LPS binds to Toll-like receptor 4 (TLR4), which is present on the plasma membrane of immune cells [4]. TLR4 is also found on the surface of renal tubular epithelial cells [6]. The binding of LPS to the receptor activates various signaling cascades, including the nuclear factor-κB (NF-κB) and mitogen-activated protein kinase (MAPK) pathways [2]. Immune cell infiltration also contributes to the overproduction of pro-inflammatory mediators [2]. In addition, the exposure of LPS to the renal tubular epithelium results in an increase in oxidative injury and apoptotic cell death [7,8].

Natural products have long been used to treat a wide range of human diseases and are now recognized as important sources of therapeutic agents [9]. Their rich structural diversity and complexity provide a wider biologically relevant chemical space. Among them, flavonoids are a group of natural phenolic compounds that are found in fruits, vegetables, and Chinese herbal medicine [10]. Flavonoids are known to possess various biological activities, including anti-inflammatory, antioxidant, and anti-apoptotic activities. Recent animal studies have shown that some flavonoids, such as fisetin [11], rutin [12], and baicalin [13], have protective effects in septic AKI. Hispidulin is a natural flavonoid commonly found in a wide range of plants, including *Grindelia argentina*, *Arrabidaea chica*, and *Saussurea involucrate* [14,15]. Previous studies have shown that this compound has versatile biological activities, including anti-cancer, anti-inflammatory, and antioxidant activities [14,15]. Hispidulin administration has been shown to ameliorate skin inflammation [16,17], cerebral ischemia-reperfusion injury [18,19], cardiac hypertrophy [20], osteoporosis [21,22], and drug-induced hepatotoxicity [23] in rodents. Moreover, hispidulin has been shown to exert an anti-inflammatory effect in LPS-treated microglial cells [24]. However, the effect of hispidulin on LPS-induced AKI has not been evaluated. The present study aimed to investigate the potential therapeutic effect of hispidulin in endotoxin-injected mice.

## 2. Results

### 2.1. Hispidulin Ameliorated LPS-Induced Renal Dysfunction

**Figure 1:**
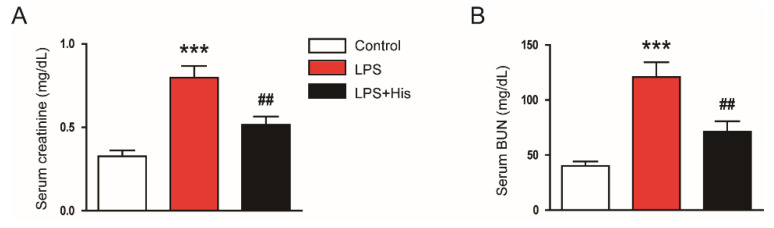
Effect of hispidulin on kidney function. (**A**) Serum creatinine concentrations. (**B**) Serum BUN concentrations. *n* = 8 in each group. *** *p* < 0.001 vs. control. ^##^ *p* < 0.01 vs. LPS.

To assess renal function, the serum concentrations of creatinine and blood urea nitrogen (BUN), two widely used indicators of kidney function [25], were measured. As shown in Figure 1A,B, hispidulin administration significantly decreased the serum levels of creatinine (LPS, 0.80 ± 0.07 mg/dL vs. LPS *+* His, 0.52 ± 0.05 mg/dL, *p* < 0.01) and BUN (LPS, 120.8 ± 13.5 mg/dL vs. LPS + His, 71.1 ± 9.6 mg/dL, *p* < 0.01).

### 2.2. Hispidulin Alleviated LPS-Induced Histological Abnormalities

**Figure 2:**
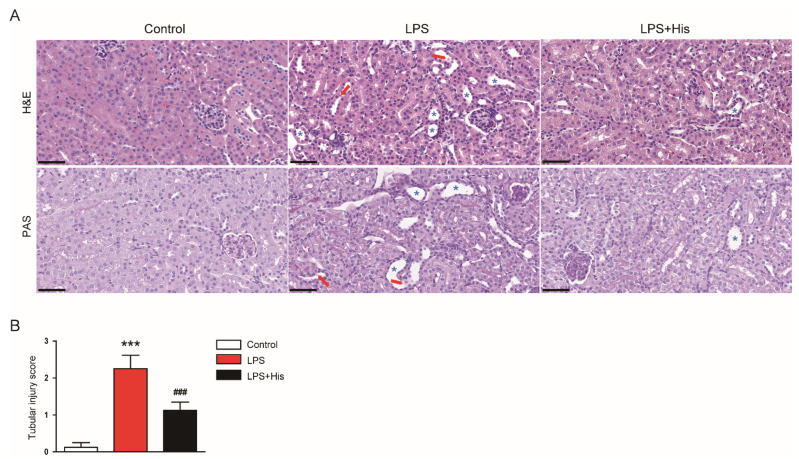
Effect of hispidulin on histological alterations. (**A**) H&E and PAS staining. Scale bar: 50 μm. Red arrows indicate detached tubular cells. Blue asterisks indicate dilated tubules. (**B**) Semi-quantitative tubular injury score of kidney sections. *n* = 8 in each group. *** *p* < 0.001 vs. control. ^#^^##^ *p* < 0.001 vs. LPS.

Hematoxylin and eosin (H&E) and periodic acid-Schiff (PAS) staining revealed that LPS injection induced histological alterations, such as tubular dilatation and tubular cell detachment, especially in the proximal tubules; these histopathological changes were significantly attenuated by hispidulin (tubular injury score: LPS, 2.3 ± 0.4 vs. LPS + His, 1.1 ± 0.2, *p* < 0.001; Figure 2A,B).

**Figure 3:**
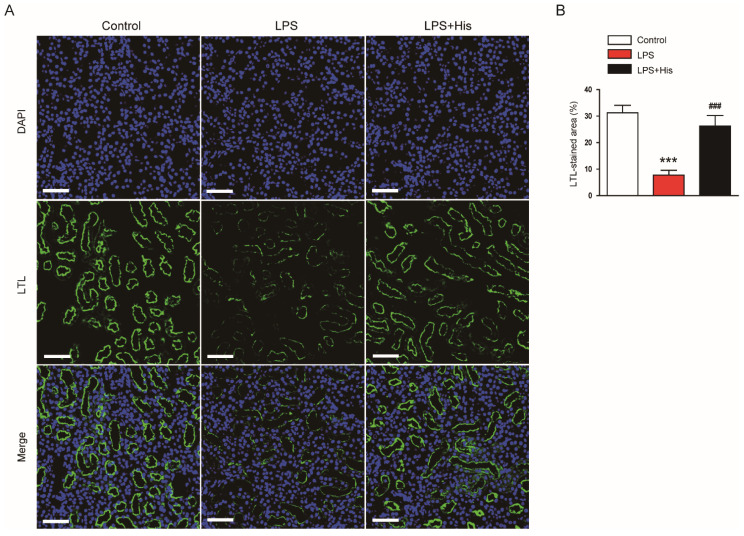
Effect of hispidulin on brush border loss in the tubules. (**A**) IF staining of LTL (green: LTL; blue: DAPI) on kidney sections. Scale bar: 60 μm. (**B**) Quantitative data of LTL-positive area. *n* = 8 in each group. *** *p* < 0.001 vs. control. ^#^^##^ *p* < 0.001 vs. LPS.

Immunofluorescence (IF) staining for lotus tetragonolobus lectin (LTL), a marker of the tubule brush border [26], revealed that LPS injection markedly induced brush border loss in the tubules, and hispidulin administration significantly reversed these changes (LPS, 7.8 ± 1.8 % vs. LPS + His, 26.3 ± 4.0 %, *p* < 0.001; Figure 3A,B).

**Figure 4:**
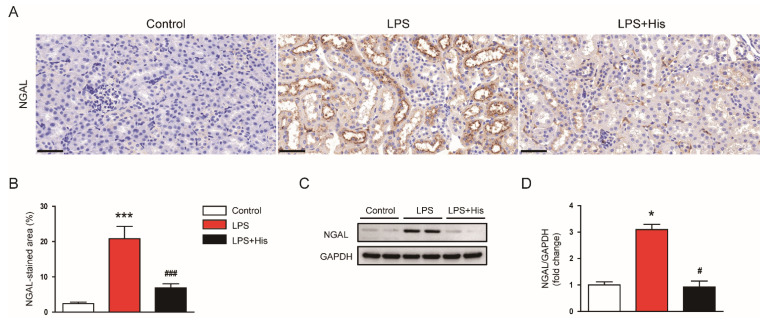
Effect of hispidulin on the expression of NGAL. (**A**) NGAL immunostaining on kidney sections. Scale bar: 50 μm. (**B**) Quantitative data of NGAL-positive area. (**C**) Western blot bands of NGAL. (**D**) Quantification results from (**C**). *n* = 8 in each group. * *p* < 0.05 and *** *p* < 0.001 vs. control. ^#^ *p* < 0.05 and ^#^^##^ *p* < 0.001 vs. LPS.

Neutrophil gelatinase-associated lipocalin (NGAL) is useful as a tubular injury marker [27,28]. An immunohistochemistry (IHC) study showed that LPS injection increased NGAL expression in the kidney (Figure 4A,B). However, hispidulin administration significantly attenuated the upregulation of NGAL (LPS, 20.9 ± 3.5 % vs. LPS + His, 6.9 ± 1.2 %, *p* < 0.001; Figure 4A,B). This result was confirmed by Western blotting (LPS, 3.1 ± 0.2 vs. LPS + His, 0.9 ± 0.2, *p* < 0.05; Figure 4C,D).

### 2.3. Hispidulin Inhibited LPS-Induced Inflammation

**Figure 5:**
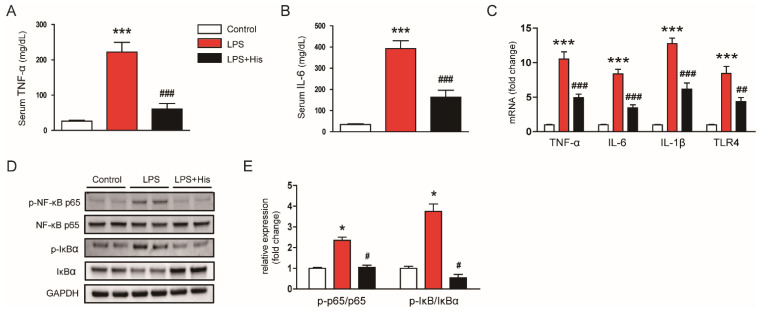
Effect of hispidulin on cytokine levels and the NF-κB pathway. (**A**) Serum TNF-α concentrations. (**B**) Serum IL-6 concentrations. (**C**) Relative mRNA levels of TNF-α, IL-6, IL-1β, and TLR4 in kidney tissues. (**D**) Western blot bands of p-NF-κB p65 and p-IκBα. (**E**) Quantification results from (**D**). *n* = 8 in each group. * *p* < 0.05 and *** *p* < 0.001 vs. control. ^#^ *p* < 0.05, ^#^^#^ *p* < 0.01 and ^#^^#^^#^ *p* < 0.001 vs. LPS.

Endotoxin injection also increased serum tumor necrosis factor-α (TNF-α) and interleukin-6 (IL-6) concentrations; these increases were significantly alleviated by hispidulin administration (TNF-α: LPS, 222.4 ± 27.2 mg/dL vs. LPS + His, 60.8 ± 15.3 mg/dL, *p* < 0.001; IL-6: LPS, 392.6 ± 37.2 mg/dL vs. LPS + His, 162.9 ± 32.7 mg/dL, *p* < 0.001; Figure 5A,B). Real-time reverse transcription polymerase chain reaction (RT-PCR) analysis also showed that LPS injection upregulated the renal mRNA levels of TNF-α, IL-6, IL-1β, and TLR4; this upregulation was significantly attenuated by hispidulin administration (TNF-α: LPS, 10.5 ± 1.0 vs. LPS + His, 4.9 ± 0.5, *p* < 0.001; IL-6: LPS, 8.4 ± 0.7 vs. LPS + His, 3.5 ± 0.4, *p* < 0.001; IL-1β: LPS, 12.8 ± 0.8 vs. LPS + His, 6.2 ± 0.9, *p* < 0.001; TLR4: LPS, 8.4 ± 1.0 vs. LPS + His, 4.4 ± 0.6, *p* < 0.01; Figure 5C). In addition, LPS injection increased p-NF-κB p65 and p-IκBα levels, while decreasing IκBα levels (Figure 5D,E). However, hispidulin administration significantly attenuated the activation of the NF-κB cascade (p-NF-κB p65: LPS, 2.4 ± 0.2 vs. LPS + His, 3.8 ± 0.4, *p* < 0.05; p-IκBα: LPS, 3.8 ± 0.4 vs. LPS + His, 0.6 ± 0.2, *p* < 0.05; Figure 5D,E).

**Figure 6:**
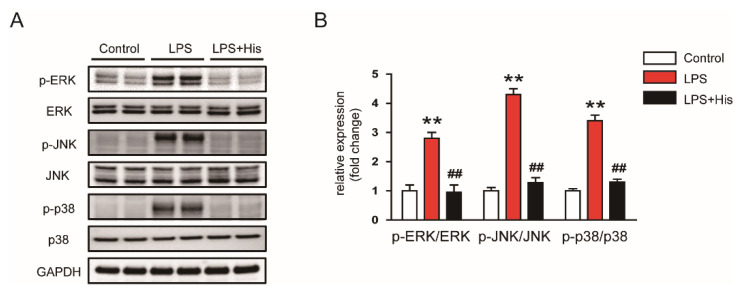
Effect of hispidulin on the MAPK pathway. (**A**) Western blot bands of p-ERK, p-JNK, and p-p38 in kidney tissues. (**B**) Quantification results from (**A**). *n* = 8 in each group. ** *p* < 0.01 vs. control. ^#^^#^ *p* < 0.01 vs. LPS.

The MAPK pathway plays a critical role in modulating inflammatory responses during sepsis [29,30]. We found that endotoxin injection increased the occurrence of the phosphorylated forms of extracellular signal-regulated kinase (ERK), c-Jun *N*-terminal kinase (JNK), and p38 in kidney tissues, which was significantly suppressed by hispidulin administration (p-ERK: LPS, 2.8 ± 0.2 vs. LPS + His, 1.0 ± 0.3, *p* < 0.01; p-JNK: LPS, 4.3 ± 0.2 vs. LPS + His, 1.3 ± 0.2, *p* < 0.01; p-p38: LPS, 3.4 ± 0.2 vs. LPS + His, 1.3 ± 0.1, *p* < 0.01; Figure 6A,B).

**Figure 7:**
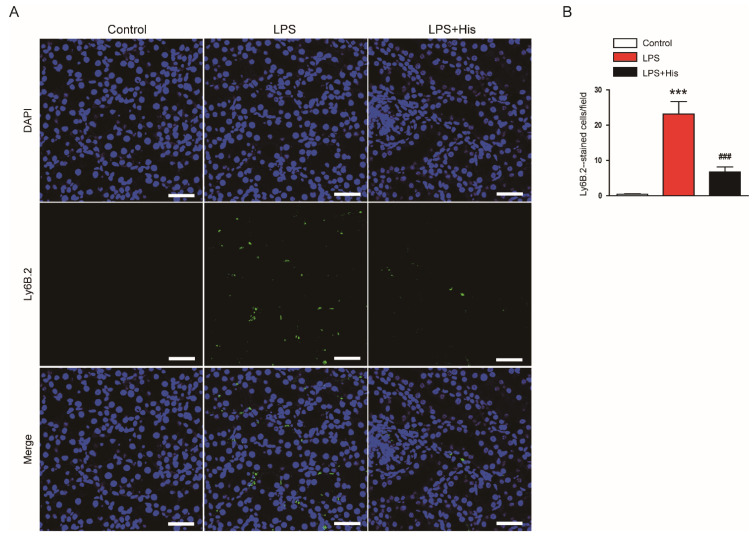
Effect of hispidulin on neutrophil infiltration. (**A**) IF staining of Ly6B.2 (green: Ly6B.2; blue: DAPI) on kidney sections. Scale bar: 40 μm. (**B**) Quantitative data of Ly6B.2-positive cells. *n* = 8 in each group. *** *p* < 0.001 vs. control. ^#^^#^^#^ *p* < 0.001 vs. LPS.

IF staining for Ly6B.2, a neutrophil marker [31,32], was performed to identify neutrophils infiltrating the kidney tissue. LPS injection increased the number of Ly6B.2-positive cells (Figure 7A,B). However, hispidulin administration significantly attenuated neutrophil infiltration (LPS, 23.1 ± 3.6 vs. LPS + His, 6.8 ± 1.4, *p* < 0.001; Figure 7A,B).

**Figure 8:**
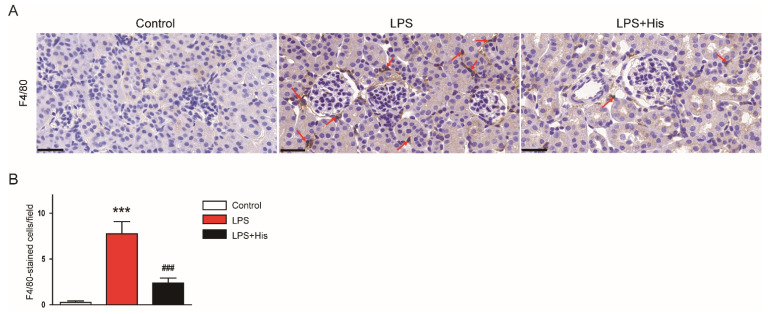
Effect of hispidulin on macrophage infiltration. (**A**) F4/80 immunostaining on kidney sections. Red arrows indicate positive cells. Scale bar: 30 μm. (**B**) Quantitative data of F4/80-positive cells. *n* = 8 in each group. *** *p* < 0.001 vs. control. ^#^^#^^#^ *p* < 0.001 vs. LPS.

IHC staining with an antibody against F4/80, a macrophage marker [33], also revealed that LPS injection increased the number of F4/80-positive cells in kidney tissues, which was significantly alleviated by hispidulin administration (LPS, 7.8 ± 1.3 vs. LPS + His, 2.4 ± 0.5, *p* < 0.001; Figure 8A,B).

### 2.4. Hispidulin Suppressed LPS-Induced Oxidative Stress

**Figure 9:**
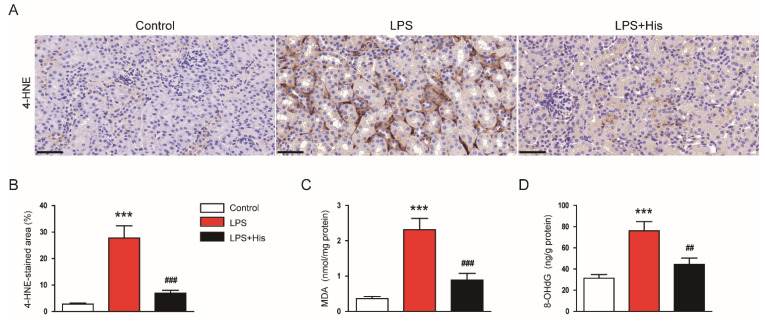
Effect of hispidulin on oxidative stress. (**A**) 4-HNE immunostaining on kidney sections. Scale bar: 50 μm. (**B**) Quantitative data of 4-HNE-positive area. (**C**) MDA levels. (**D**) 8-OHdG levels. *n* = 8 in each group. *** *p* < 0.001 vs. control. ^#^^#^ *p* < 0.01 and ^#^^##^ *p* < 0.001 vs. LPS.

The amounts of 4-hydroxynonenal (4-HNE) and malondialdehyde (MDA), two lipid peroxidation products [34,35], in the kidney tissues were analyzed to assess oxidative stress. IHC staining for 4-HNE showed that LPS injection largely increased 4-HNE expression in kidney tissues, and hispidulin administration significantly alleviated this change (LPS, 27.8 ± 4.6 % vs. LPS + His, 6.9 ± 1.2 %, *p* < 0.001; Figure 9A,B). Renal MDA levels were also elevated after endotoxin injection; this effect was largely attenuated by hispidulin (LPS, 2.3 ± 0.3 nmol/mg protein vs. LPS + His, 0.9 ± 0.2 nmol/mg protein, *p* < 0.001; Figure 9C). Moreover, the renal levels of 8-hydroxy-2′-deoxyguanosine (8-OHdG), a DNA oxidation marker [36,37], were elevated after LPS injection (Figure 9D). However, hispidulin administration significantly reversed this increase in 8-OHdG levels (LPS, 76.1 ± 8.6 ng/g protein vs. LPS + His, 44.3 ± 6.1 ng/g protein, *p* < 0.001; Figure 9D).

**Figure 10:**
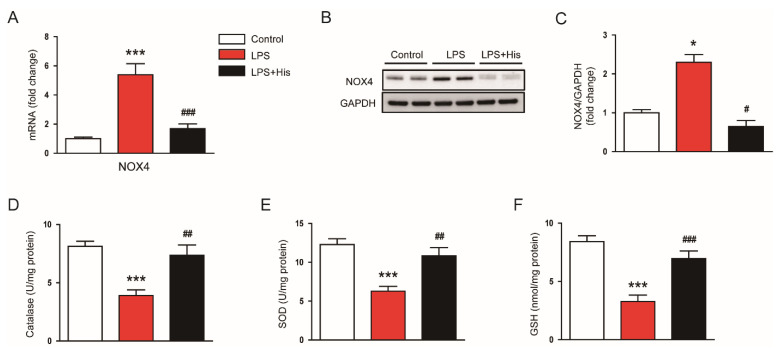
Effect of hispidulin on pro-oxidant and antioxidant systems. (**A**) Relative mRNA level of NOX4 in kidney tissues. (**B**) Western blot bands of NOX4. (**C**) Quantification results from (**B**). (**D**) Catalase activity. (**E**) SOD activity. (**F**) GSH levels in kidney tissues. *n* = 8 in each group. * *p* < 0.05 and *** *p* < 0.001 vs. control. ^#^ *p* < 0.05, ^#^^#^ *p* < 0.01, and ^#^^##^ *p* < 0.001 vs. LPS.

To examine the potential mechanisms underlying the antioxidant effect of hispidulin, changes in pro-oxidant and antioxidant systems were investigated. As shown in Figure 10A, the mRNA expression of NADPH oxidase 4 (NOX4) was increased after LPS injection, and this upregulation was significantly alleviated by hispidulin administration (LPS, 5.4 ± 0.8 vs. LPS + His, 1.7 ± 0.3, *p* < 0.001). This result was also confirmed by Western blotting (LPS, 2.3 ± 0.2 vs. LPS + His, 0.7 ± 0.2, *p* < 0.05; Figure 10B,C). In addition, catalase and superoxide dismutase (SOD) activities were decreased in kidney tissues after LPS injection; these decreases were also significantly attenuated by hispidulin administration (catalase: LPS, 3.9 ± 0.5 U/mg protein vs. LPS + His, 7.4 ± 0.9 U/mg protein, *p* < 0.01; SOD: LPS, 6.3 ± 0.6 U/mg protein vs. LPS + His, 10.8 ± 1.1 U/mg protein, *p* < 0.01; Figure 10D,E). Hispidulin administration also significantly reversed the decrease in renal glutathione (GSH) content in LPS-injected mice (LPS, 3.3 ± 0.5 nmol/mg protein vs. LPS + His, 7.0 ± 0.7 nmol/mg protein, *p* < 0.001; Figure 10F).

### 2.5. Hispidulin Attenuated LPS-Induced Apoptosis

**Figure 11:**
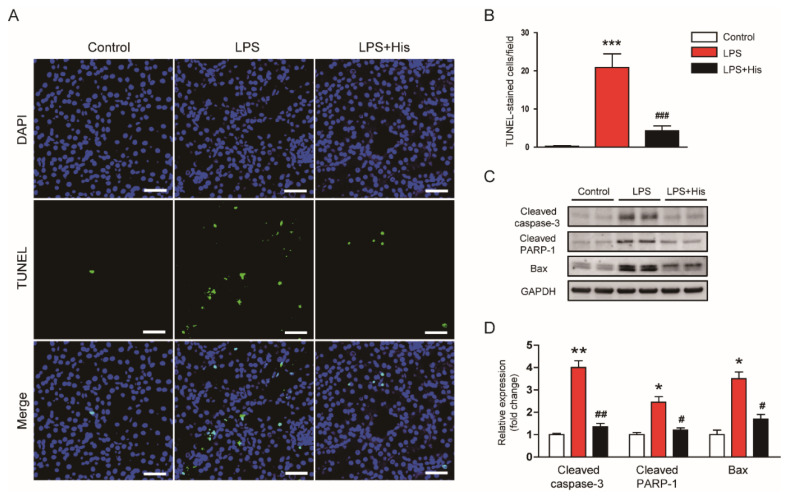
Effect of hispidulin on apoptosis. (**A**) TUNEL staining (green: TUNEL; blue: DAPI). Scale bar: 60 μm. (**B**) Quantitative data of TUNEL-positive cells. (**C**) Western blot bands of cleaved caspase-3, cleaved PARP-1, and Bax. (**D**) Quantification results from (**C**). *n* = 8 in each group. * *p* < 0.05, ** *p* < 0.01 and *** *p* < 0.001 vs. control. ^#^ *p* < 0.05, ^#^^#^ *p* < 0.01, and ^#^^#^^#^ *p* < 0.001 vs. LPS.

A TdT-mediated dUTP nick end labeling (TUNEL) assay was performed to evaluate the effect of hispidulin on tubular cell apoptosis in kidney tissues. As shown in Figure 11A,B, endotoxin injection increased the number of TUNEL-positive cells; this effect was significantly alleviated by hispidulin administration (LPS, 20.9 ± 3.6 vs. LPS + His, 4.3 ± 1.3, *p* < 0.001). LPS injection also increased the protein levels of cleaved caspase-3, cleaved poly(ADP-ribose) polymerase-1 (PARP-1), and Bax (Figure 11C,D). However, hispidulin administration significantly inhibited the caspase-3-dependent pathway in the endotoxin-injected mice (cleaved caspase-3: LPS, 4.0 ± 0.3 vs. LPS + His, 1.4 ± 0.2, *p* < 0.01; cleaved PARP-1: LPS, 2.5 ± 0.3 vs. LPS + His, 1.2 ± 0.1, *p* < 0.05; Bax: LPS, 3.5 ± 0.3 vs. LPS + His, 1.7 ± 0.2, *p* < 0.05; Figure 11C,D).

## 3. Discussion

**Figure 12:**
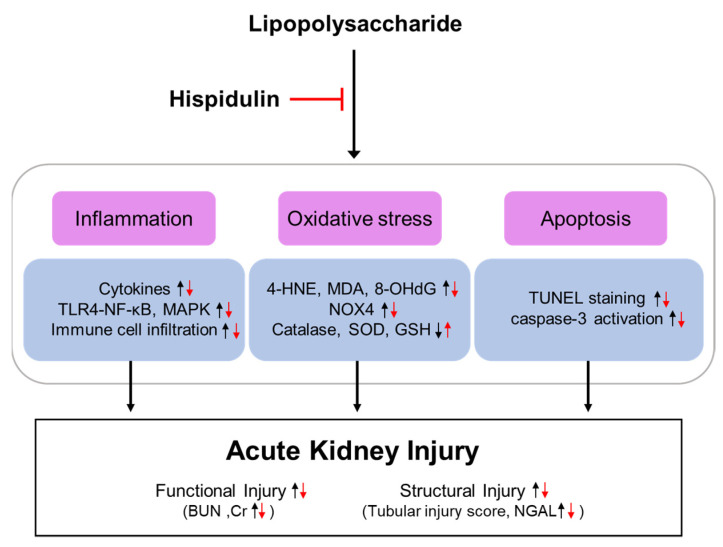
Schematic summary of the key findings of the present study. Hispidulin attenuated LPS-induced functional and structural renal injury by suppressing inflammation, oxidative stress, and apoptosis.

In this study, we aimed to investigate whether hispidulin exerts a renoprotective effect on endotoxin-induced AKI. We demonstrated that hispidulin administration ameliorated endotoxin-induced kidney injury, as evidenced by the decrease in serum creatinine and BUN levels and the improvement in renal histological abnormalities. Mechanistically, this renoprotective action of hispidulin was associated with the amelioration of inflammation, oxidative stress, and apoptosis (Figure 12).

Hispidulin is a natural flavonoid that has multiple biological activities, including anti-cancer, anti-inflammatory, and antioxidant activities [14,15]. This compound has been shown to exert beneficial effects on various inflammatory diseases [16,17,18,19,20,21,22,23]. In this study, the administration of hispidulin ameliorated functional and structural renal injury in LPS-injected mice. Serum creatinine and BUN levels were measured to assess kidney function. These indicators are widely used in animal experiments to evaluate kidney function [25,26,27,28,29,30,31]. We found that hispidulin significantly attenuated the LPS-induced renal dysfunction. In addition, hispidulin alleviated renal structural injury, as evidenced by an improvement in tubular injury score, a reduction in the loss of the brush border, and the downregulation of NGAL. NGAL is a well-known tubular injury marker, and its expression is increased in various kidney diseases [27,28]. Altogether, these results indicate that hispidulin has a protective effect against LPS-induced functional and structural injury.

The TLR4 signaling cascade is critically involved in the development and progression of inflammatory responses during sepsis [2]. Previous studies have shown that targeting TLR4 attenuates endotoxin-induced inflammation and kidney injury in animals [38,39]. TLR4 is highly expressed in immune cells and is also found in renal tubular epithelial cells [4,6]. LPS is a well-known ligand for TLR4 that stimulates the receptor to activate key signaling cascades, including the NF-κB and MAPK pathways, resulting in an overproduction of cytokines [2]. In addition, LPS induces the infiltration of immune cells into the injured tissues [40,41]. In this study, LPS injection induced an excessive production of cytokines, increased TLR4 expression, and activated the NF-κB and MAPK (ERK, JNK, and p38) cascades. Interestingly, hispidulin administration significantly attenuated these inflammatory responses. In agreement with our results, the anti-inflammatory action of hispidulin has been reported in numerous studies [15]. Importantly, a recent study showed that hispidulin reduced cytokine production and inhibited NF-κB activation in LPS-treated microglial cells [24]. These cells are the resident macrophages of the brain and express TLR4 in high amounts [42]. Thus, our findings suggest that the ameliorating effects of hispidulin on endotoxin-induced inflammatory responses may be primarily due to the suppression of TLR4-dependent signaling pathways. Moreover, we also found that hispidulin significantly inhibited the infiltration of Ly6B.2-positive neutrophils and F4/80-positive macrophages into the damaged kidneys. Ly6B.2 and F4/80 are glycoproteins that are present on the cell surface [43]. While Ly6B.2 is expressed mainly on neutrophils, F4/80 is found primarily on macrophages. The infiltration of Ly6B.2-positive or F4/80-positive cells into the damaged tissue has been shown to be increased in various inflammatory diseases, such as postoperative ileus [32], cisplatin-induced renal injury [28,31], cholestatic liver disease [33,37], nonalcoholic fatty liver disease [44], and atherosclerosis [45]. Neutrophils and macrophages may exacerbate the LPS-induced inflammatory responses [2]. Therefore, the anti-inflammatory effect of hispidulin in LPS-induced AKI can be attributed, at least in part, to its inhibitory effect on neutrophil and macrophage infiltration.

In addition to inflammation, oxidative stress and apoptosis also play important roles in LPS-induced AKI [46,47]. Numerous studies have shown that LPS-injected animals display marked oxidative stress and tubular cell apoptosis in the kidneys [46]. Hispidulin has been known to possess an antioxidant property [48]. In this study, LPS injection induced an increase in lipid and DNA oxidation products. 4-HNE and MDA are lipid peroxidation products [34,35] and 8-OHdG is a product of oxidatively damaged DNA [36,37]. These molecules have been widely used to assess oxidative stress in animal models of various human diseases, such as acetaminophen-induced liver injury [49], ischemia/reperfusion-induced myocardial injury [50], acute lung injury [51], and traumatic brain injury [52]. We found that an increase in the amount of lipid and DNA oxidation products was accompanied by the upregulation of NOX4, the inhibition of catalase and SOD activities, and a decrease in GSH content. However, all these changes were largely reversed by hispidulin. In LPS-induced AKI, NOX4 is upregulated and produces reactive oxygen species (ROS), leading to oxidative tissue damage [53]. Thus, the downregulation of NOX4 may be critically related to the suppressive effect of hispidulin on endotoxin-induced oxidative stress. Consistently, in vitro studies have shown that the compound inhibits ROS generation in inflammatory conditions [24,54]. In addition, it has been shown that antioxidant enzymes, such as catalase and SOD, play crucial roles in LPS-induced AKI [47]. GSH is an endogenous antioxidant that protects cells from oxidative cellular injury [55]. Therefore, the activation of the antioxidant system along with the inhibition of the pro-oxidant system appears to be involved in the beneficial action of hispidulin. It has been shown that nuclear factor erythroid-2-related factor 2 (Nrf2) is a key transcription factor that modulates the expression of antioxidant enzymes, including catalase and SOD [56]. Previous studies have shown that Nrf2 activation can serve as a useful therapeutic approach for septic AKI [57,58,59]. Interestingly, hispidulin can activate Nrf2-dependent antioxidant systems to suppress oxidative stress [19,60,61]. Although the effect of hispidulin on Nrf2 was not examined in the present study, it is likely that hispidulin enhanced the antioxidant system by activating Nrf2. Furthermore, hispidulin largely inhibited tubular cell apoptosis in endotoxin-injected mice, as evidenced by the decrease in TUNEL-stained cells and the reversal of caspase-3 activation. Similar to our findings, under inflammatory conditions, hispidulin has been shown to exert anti-apoptotic effects in podocytes [62] and neurons [63]. Because hispidulin can induce apoptosis in tumor cells [64], this compound appears to have differing actions between normal and tumor cells.

Although the bioavailability of hispidulin has rarely been studied, it is known that the bioavailability of flavonoids is generally low and can vary widely between individual compounds [65]. These shortcomings limit the clinical application of flavonoids despite their numerous health benefits. Therefore, much effort should be put into the development of strategies to enhance bioavailability, for example, by improving the intestinal absorption, changing the site of absorption, and improving the metabolic stability of these compounds [65,66].

## 4. Materials and Methods

### 4.1. Animal Experiments

**Figure 13:**
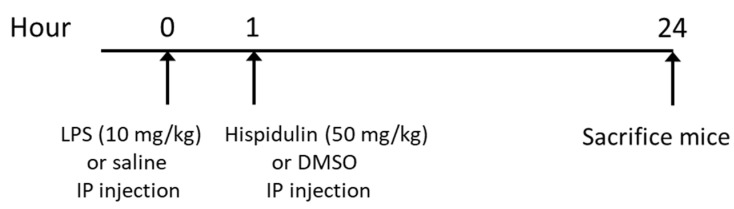
Overview of the animal experiment protocol.

Twenty-four male C57BL/6 mice (7 weeks of age) were provided by HyoSung Science (Daegu, Korea) and were caged at a temperature of 20–24 °C and humidity of 60–70% with a 12/12 h light–dark cycle. The mice were arbitrarily divided into the following groups (*n* = 8 in each group): control group, LPS group, and LPS + His group. The LPS group and the LPS + His group received a single intraperitoneal injection of LPS (10 mg/kg; dissolved in saline). One hour after LPS injection, the mice of the LPS + His group were intraperitoneally injected with hispidulin (50 mg/kg; dissolved in DMSO). LPS and hispidulin were obtained from Sigma-Aldrich (St. Louis, MO, USA). The same volumes of vehicles were given to the mice in the control group or the LPS group. The protocol is summarized in Figure 13. The dose of hispidulin was chosen based on the results of previous studies [23,67,68]. At 24 h after LPS injection, all mice were anesthetized and sacrificed. Blood and kidney samples were collected for further analyses. The animal experiments were approved by the Institutional Animal Care and Use Committee of the Daegu Catholic University Medical Center (DCIAFCR-210810-16-Y).

### 4.2. Biochemial Analyses of Serum and Kidney Tissue

The serum concentrations of creatinine and BUN were measured using a biochemical autoanalyzer (Hitachi, Osaka, Japan). Catalase and SOD activities were analyzed using colorimetric activity kits (Invitrogen, Carlsbad, CA, USA). The serum concentrations of TNF-α and IL-6 were determined using ELISA kits (R&D Systems, Minneapolis, MN, USA). MDA and 8-OHdG levels were analyzed using an MDA assay kit (Sigma-Aldrich, St. Louis, MO, USA) and an 8-OHdG assay kit (Abcam, Cambridge, MA, USA), respectively. GSH levels were determined using a GSH detection kit (Enzo Life Sciences, Farmingdale, NY, USA). All analyses were carried out following the manufacturers’ protocols.

### 4.3. Histological Examination, IHC, and IF Staining

Kidney tissues were fixed in 10% formalin, dehydrated in graded ethanol, embedded in paraffin, and stained with H&E or PAS reagent. The percentage of damaged area was evaluated to assess the severity of tubular injury as follows: 0, 0%; 1, ≤10%; 2, 11–25%; 3, 26–45%; 4, 46–75%; and 5, 76–100% [69,70,71]. The tubular injury score was based on tubular cell necrosis, the loss of brush border, cast formation, and tubular dilatation [54]. IHC staining on deparaffinized sections was carried out using primary antibodies against NGAL (Abcam), F4/80 (Santa Cruz Biotechnology, Santa Cruz, CA, USA), or 4-HNE (Abcam). Then, the sections were incubated with an HRP-conjugated secondary antibody. For IF staining, deparaffinized sections were probed with anti-Ly6B.2 primary antibody (Abcam). Then, the sections were incubated with a secondary antibody conjugated with Alexa Fluor 488 (Invitrogen). A FITC-labeled LTL (Vector Laboratories, Burlingame, CA, USA) was used for detecting the brush border of the tubules. The images were captured using a confocal microscope (Nikon, Tokyo, Japan). The quantification of positive staining was analyzed using an image-analyzing software (IMT i-Solution, Coquitlam, BC, Canada) in 10 randomized fields per sample.

### 4.4. Western Blot

Western blot analysis was performed as previously described [72]. Kidney tissues were lysed in a lysis buffer (Cayman Chemical, Ann Arbor, MI, USA). Protein concentrations were determined using a BCA protein assay kit (Bio-Rad Laboratories, Hercules, CA, USA). Protein samples were loaded onto precast gradient polyacrylamide gels (Thermo Fisher Scientific, Waltham, MA, USA) and then transferred to a nitrocellulose membrane. The membranes were incubated with primary antibodies against NGAL, NF-κB p65, p-NF-κB p65, IκBα, p-IκBα, p-ERK, ERK, p-JNK, JNK, p-p38, p38, NOX4, cleaved caspase-3, cleaved PARP-1, Bax, or glyceraldehyde-3-phosphate dehydrogenase (GAPDH). All primary antibodies were acquired from Cell Signaling Technology (Danvers, MA, USA), except for the antibodies against NGAL (Santa Cruz Biotechnology, Santa Cruz, CA, USA), NOX4 (Novus Biologicals, Littleton, CO, USA), and Bax (Santa Cruz Biotechnology, Santa Cruz, CA, USA). Then, the membranes were probed with an HRP-conjugated secondary antibody. The blots were visualized and quantified using enhanced chemiluminescence reagents (Millipore, Bedford, MA, USA) and the iBright CL1500 Imaging System (Thermo Fisher Scientific, Waltham, MA, USA).

### 4.5. Real-Time RT-PCR

The tissues were dissolved in TRIzol reagent (Sigma-Aldrich). Extracted RNA was reverse transcribed into cDNA using the PrimeScript RT Reagent Kit (Takara, Tokyo, Japan). Real-time RT-PCR was performed using the LightCycler^®^ 480 SYBR Green I Master (Roche Diagnostics, Indianapolis, IN, USA) and specific primers (Table 1) in a Thermal Cycler Dice Real Time System III (Takara). Data were calculated using the 2^−ΔΔCT^ method and normalized to GAPDH levels.

### 4.6. TUNEL Assay

Apoptotic cells were identified in kidney sections using a TUNEL assay kit (Roche Diagnostics, Indianapolis, IN, USA) following the manufacturer′s instructions. Briefly, the sections were deparaffinized in xylene, rehydrated using descending grades of ethanol, and permeabilized for 30 min at room temperature with proteinase K in 10 mM Tris-HCl, pH 7.4–8. After washing, the sections were incubated in the TUNEL reaction mixture for 1 h at 37 °C. Nuclei were counterstained with DAPI. The images were captured using a confocal microscope (Nikon, Tokyo, Japan). Positive cells were analyzed in 10 randomized fields per sample.

### 4.7. Statistical Analysis

Data were expressed as the mean ± SEM. Because the data followed a Gaussian distribution when assessed with the Kolmogorov–Smirnov test, statistical significance was determined using a one-way ANOVA analysis and Bonferroni’s multiple comparison tests. All statistical analyses were assessed using SPSS version 25.0 (IBM Corp., Armonk, NY, USA). A *p*-value less than 0.05 was considered significant.

## 5. Conclusions

In conclusion, the current study demonstrated that hispidulin has a renoprotective effect on endotoxin-induced AKI. Hispidulin alleviated TLR4-dependent cytokine production and immune cell infiltration, inhibited oxidative stress via the modulation of pro-oxidant and antioxidant enzymes, and attenuated tubular cell apoptosis along with inhibition of caspase-3 activation. Although previous studies have reported that hispidulin has various biological activities, the potential effect of the compound on septic AKI has not been determined. Our findings suggest that this natural flavonoid might be a potential therapeutic option for septic AKI.

## Figures and Tables

**Table 1:** List of primers.

Gene	Primer Sequence (5′→3′)	Accession No.
TNF-α	Forward: CACAGAAAGCATGATCCGCGACGT Reverse: CGGCAGAGAGGAGGTTGACTTTCT	NM_013693
IL-6	Forward: TAGTCCTTCCTACCCCAATTTCC Reverse: TTGGTCCTTAGCCACTCCTTC	NM_031168
IL-1β	Forward: CGCAGCAGCACATCAACAAGAGC Reverse: TGTCCTCATCCTGGAAGGTCCACG	NM_008361
TLR4	Forward: CCTGACACCAGGAAGCTTGAA Reverse: TCTGATCCATGCATTGGTAGGT	NM_021297
NOX4	Forward: CCCAAGTTCCAAGCTCATTTCC Reverse: TGGTGACAGGTTTGTTGCTCCT	NM_015760
GAPDH	Forward: ACTCCACTCACGGCAAATTC Reverse: TCTCCATGGTGGTGAAGACA	NM_001289726

## Data Availability

The data supporting the findings of this study are available within the article.

## Sample Availability

Samples of the compounds are not available from the authors.
